# Patient and public involvement in stroke research: a scoping review protocol

**DOI:** 10.12688/hrbopenres.13449.2

**Published:** 2022-07-28

**Authors:** Patricia Hall, Thilo Kroll, Julianne Hickey, Diarmuid Stokes, Olive Lennon

**Affiliations:** 1 iPASTAR Collaborative Doctoral Award Programme, RCSI Division of Population Health Sciences, RCSI University of Medicine and Health Sciences, Dublin 2, Dublin, D02 H903, Ireland; 2 School of Public Health, Physiotherapy and Sports Science, Health Science Centre, University College Dublin, Belfield, Dublin 4, D04 C7X2, Ireland; 3 UCD Centre for Interdisciplinary Research, Education and Innovation in Health Systems (UCD IRIS), University College Dublin, Belfield, Dublin 4, D04 C7X2, Ireland; 4 UCD School of Nursing, Midwifery and Health Systems, University College Dublin, Belfield, Dublin 4, D04 C7X2, Ireland; 5 HRB PPI Ignite, University College Dublin, Belfield, Dublin 4, Ireland; 6 iPASTAR PPI Champion, Dublin, Ireland; 7 UCD Library, University College Dublin, Belfield, Dublin 4, Ireland

**Keywords:** patient and public involvement, patient engagement, patient participation, stakeholder involvement, stroke research

## Abstract

**Background: **Growing consensus supports public and patient involvement (PPI) in research as the lived experience of patients, family carers and users of health and social care services bring unique insights to healthcare research. The impact and burden of stroke present ongoing challenges for those living with its consequences and could potentially limit PPI activity. This review aims to explore PPI in published stroke research to identify and describe the extent, nature and design of PPI activities, the type/s of studies involved and the profile of PPI participants engaged in stroke research.

**Methods:** This systematic scoping review, guided by the Arksey & O’Malley five step framework, will be reported according to the PRISMA-ScR reporting guidelines. PPI is embedded at each stage of this proposed scoping review from conceptualisation, participation, contribution and collaboration. The Population, Concept, Context (PCC) structure defines the research question which asks - How is PPI in stroke research currently being conducted and how do the study authors report their PPI activities and its impact? A comprehensive range of electronic databases including PubMed, CINAHL, EMBASE, PsychINFO and the Cochrane Database of Systematic Reviews will generate a broad range of studies. Grey literature (e.g. OpenGrey, Lenus) and internationally recognised stroke organisation websites will be searched for additional research reports. Data extraction will adhere to the Joanna Briggs Institute guidelines, with results collated and mapped to the research cycle stage/s.

**Conclusion**s: The outlined scoping review protocol will comprehensively identify and map the existing scientific literature that reports PPI in stroke research. Findings will be presented in relation to PPI conceptualisation, participant profiles and activities in stroke research, volume, type and range of approaches. Knowledge gaps may be identified thereby offering opportunities and recommendations for future priorities for PPI in stroke research.

## Introduction

Stroke is a major cause of death and disability worldwide and many survivors live with significant disability ^1^. Globally over 15 million people have a stroke each year making it the second leading cause of death worldwide and leading cause of adult disability, accounting for almost 5% of disability-adjusted life years ^2, 3^. According to the latest National Audit of Stroke in Ireland (NOCA), almost 6,000 people were admitted to hospital in 2019 and 71% of those with ischaemic stroke had disabilities on discharge ^4^.

Despite advances in prevention, early recognition/diagnosis and treatment, projections indicate a significant increase in stroke events worldwide in the coming years ^5^. Whilst death rates have reduced, the burden of stroke for those living with the consequences, both survivors and their loved ones, present ongoing daily challenges. Communication difficulties, cognitive impairment, perception issues, emotional factors and general fatigue, although less obvious than physical disabilities can be equally as devastating. This burden of stroke reveals a vulnerability for people to become marginalised, limiting their ability to actively engage in their own care and/or fully participate in life after stroke ^6, 7^. The Stroke Action Plan for Europe identifies and aims to address the challenges facing stroke survivors and families, associated with life after stroke ^5^.

In healthcare research specifically, patients with a lived experience of disease, family carers and users of health and social care services bring unique insights. Over the past 20 – 30 years patient and public involvement (PPI) in health and social research has evolved and gained widespread support. The UK has been at the forefront of establishing policy support through the National Institute for Health Research (NIHR) advisory group, INVOLVE, and defines PPI as “research being carried out 'with' or 'by' members of the public rather than 'to', 'about' or 'for' them” ^8^. The term ‘public’ is used to include patients, potential patients, carers, and anyone who uses health and social care services or represents service users. In Ireland, since 2017 the Health Research Board (HRB) and the Irish Research Council (IRC) have committed to develop and support PPI with the establishment of the PPI Ignite network ^9^. PPI in research is considered to occur when “individuals meaningfully and actively collaborate” at one or more stages of the research process ^10^. The conceptual model often used to describe this collaboration is drawn from Arnstein’s ladder of citizen participation ^11^. In this model three approaches to involvement are described – consultation, collaboration, and user-control along a spectrum of involvement which can vary at different stages.

A number of arguments have been postulated for actively involving patients and the public in research. These generally relate to the political mandate for inclusion from research funders; the moral argument that supports the rationale that people affected by the outcomes of research should be included in the decision making; and the consequentialist argument that asserts the benefits to the quality of research as a result of involving service users. This latter argument has a growing consensus as the positive impact on improving research quality and strengthening relevance is acknowledged ^12^. However, there remains ambiguity in the literature on the concept and understanding of what is and what is not PPI in research ^13^. This can lead to misunderstanding and misinterpretation of the extent of public involvement in research and potential tokenistic representation especially in relation to seldom heard voice groups including individuals with stroke and their carers/family.

Adopting the four values of respect, openness, reciprocity and flexibility, and working collaboratively across all stages of involvement is recommended to support inclusivity, particularly with diverse, seldom heard groups ^14^. The impact and burden of stroke on the individual and family could potentially limit PPI activity but consideration of these barriers and facilitators has been found to benefit stroke survivors, carers and the research process ^15^. Working with people affected by stroke and health care professionals, the UK Stroke Association has identified priority areas for research across the two main stroke care pathways - Stroke prevention, diagnosis, pre-hospital and hospital care; and Stroke rehabilitation and long-term care ^16^. Stroke survivors/carers represented over 50% of contributors suggesting an interest and eagerness to be involved in the research process despite limitations.

As PPI gains recognition and importance in stroke research in principle, it is critical to understand what is happening in parallel in stroke research practice. This paper describes the protocol for a scoping review collating and commenting on current PPI practices described and reported in stroke research. To our knowledge no current scoping review has examined the published literature to explore PPI in stroke research as we outline here.

## Protocol

### Aims and objectives

*The overarching aim of this review is to identify and describe current practices of PPI in stroke research. Specifically, this review will focus on the nature, design and type/s of studies that involve patients and/or members of the public in the planning, conduct and/or dissemination of stroke research, and explore how PPI has been conceptualised in stroke research.*

Objectives:

1. map the volume, type and range of PPI approaches in stroke research
2. profile PPI participants and examine representativeness of participants from an equality, diversity and inclusion (EDI) perspective
3. map PPI research activities against the stroke research cycle
4. collate reported enablers and barriers to PPI application in stroke research
5. explore the impact of PPI on stroke research, clinical practice and health policy

### Design

A scoping review will be conducted as it is considered the most appropriate methodology to broadly map the key sources and types of evidence available when the extent and nature of the research is largely unknown ^17^. The review will conform to the 5 stages of the Arksey & O'Malley framework for scoping studies ^18^ refined by Levac ^19^ and the methodological framework described by Peters *et al.* in the JBI Manual for Evidence Synthesis (Chapter 11) ^20^. The review will be reported according to the PRISMA-ScR reporting guidelines ^21^.

Stage 1: Identify the research question

Stage 2: Identify the relevant studies

Stage 3: Study selection

Stage 4: Charting the data

Stage 5: Collating, summarising and reporting the results

Stage 6: Stakeholder consultation (optional)

**Figure 1.**
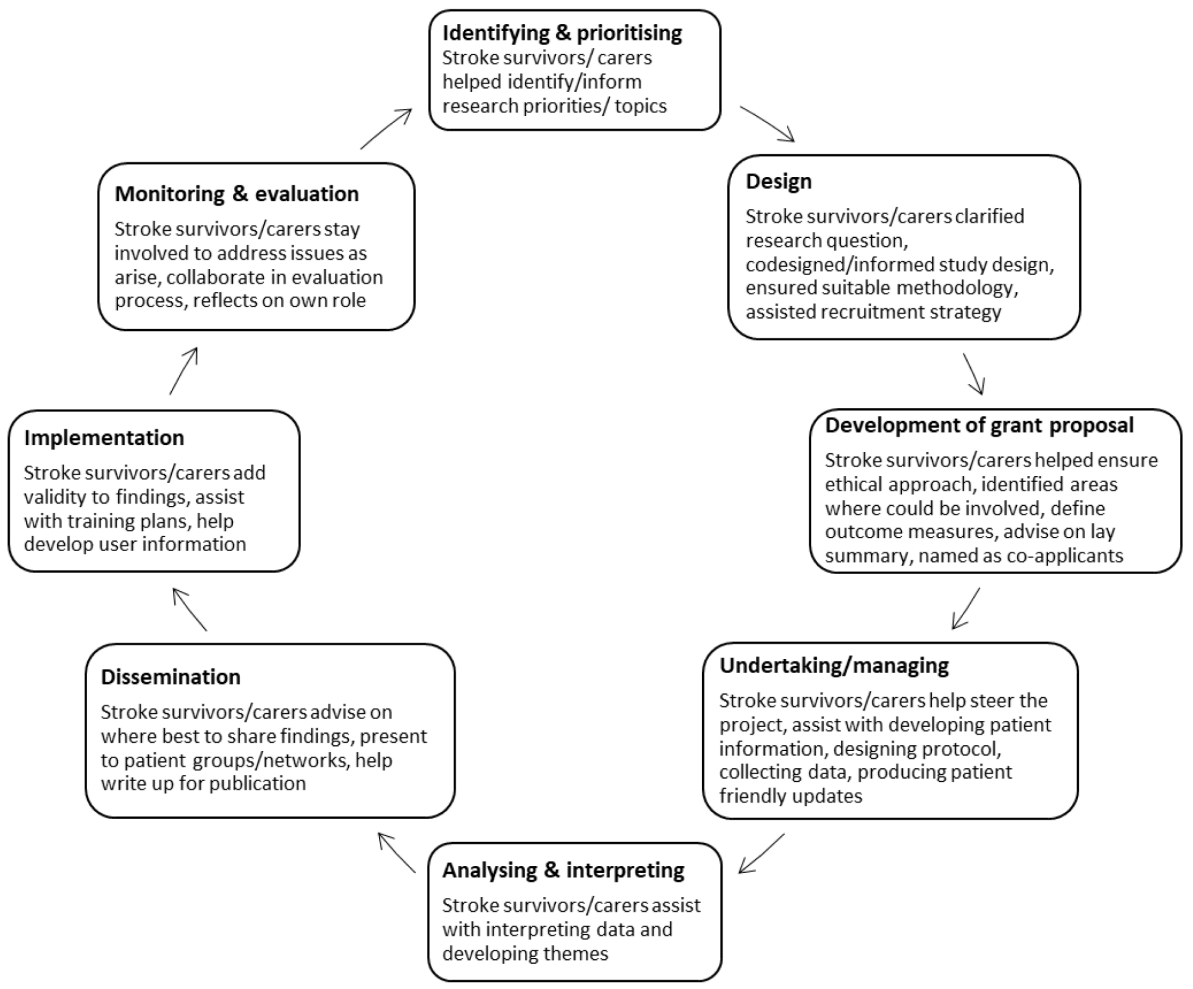
Research cycle framework ^9, 22^.

Data will be extracted and mapped to the published stages of PPI engagement across the research cycle ( Figure 1) ^9, 22^.

### Stage 1: Identify the research question

The first stage of the protocol clearly identifies the research question based on the overall aim of the scoping review; namely to examine the extent, range, and nature of stroke research that actively involves patients and the public across any or multiple stages of the research process.

The Population, Concept, Context (PCC) structure proposed by the Joanna Briggs Institute (JBI) ^23^, outlined below, was used to define the research question which asks: How is PPI in stroke research currently being conducted and how do the study authors report the details of their PPI activities and its impact?

Population: individuals who have experienced stroke. This includes stroke survivors, carers and family who can all be affected by stroke. The lived experience of each can bring unique insights to stroke research.

Concept: PPI (as defined by NIHR ^8^ as “research carried out ‘with’ or ‘by’ members of the public rather than ‘to’, ‘about’ or ‘for’ them” and where “an active partnership between patients and the public and researcher” is present). This will include all initiatives or activities irrespective of the terminology used, where there is explicit involvement of PPI partners across any of the phases of stroke research ( Figure 1).

Context: Stroke research – relating to aspects including but not limited to stroke recognition, primary or secondary prevention, acute care, treatment/management, rehabilitation, survival, long-term outcomes/care, community support.

Based on the review question, aims and objectives and PCC framework the following Inclusion/exclusion criteria was developed ( Table 1):

**Table 1.** Inclusion/exclusion criteria.

Inclusion	Exclusion
Empirical stroke research studies of any study design, including qualitative, quantitative and mixed methods and where relevant, published pre-trial consultation processes	
Studies with a clear statement in relation to PPI activities/ initiatives which fit the principles of PPI in stroke research, irrespective of terminology An inclusive approach to screening will be taken whereby if PPI is not mentioned in the title or abstract, the full text manuscript will be reviewed with particular focus on the methods and acknowledgements sections	Studies, including those where the focus is on participation or engagement in trials or other research, that do not explicitly state involvement of PPI partners in one or more stages of the stroke research cycle identified in Figure 1.
Title and abstract in English language	
A publication date cut off of 2014 will be applied to include studies published after the first handbook for health researchers was published ^22^	
Publications which include empirical data (e.g. qualitative, quantitative, meta-analyses, review papers)	Publications that do not report original empirical data (e.g. editorials, commentary pieces) and conference abstracts

### Stage 2: Identify the relevant studies

As the research question is broad, a comprehensive range of electronic databases has been identified by the authors to assist in a systematic and targeted search strategy. All publications that meet the inclusion criteria identified will be selected. No date, location or language limitations will be applied with respect to the manuscripts selected. The search will involve the electronic databases PubMed, CINAHL, EMBASE, PsycINFO and the Cochrane Database of Systematic Reviews (Extended data ^23^). These libraries were chosen as they will yield a broad, cross disciplinary range of studies. In addition, reference lists of included studies will be checked. To target relevant grey literature, a systematic search will be conducted in the key databases, e.g. OpenGrey and Lenus. A grey literature checklist e.g. Cadth (Canada’s drug and health technology agency) ^24^, will help to ensure a comprehensive search is completed. Websites relating to internationally recognised stroke organisations (e.g. World Stroke Organisation, European Stroke Organisation, UK Stroke Association, American Heart Association/ American Stroke Association and Stroke Foundation-Australia) and other charitable/non-governmental organisations (e.g. James Lind Alliance) will be searched for additional, non-indexed published research reports and policy documents which include empirical data.

The targeted search strategy, developed in consultation with the information scientist (librarian), will be adapted for each database. The key search concepts resulting from the PCC framework are ‘individuals with experience of stroke’ (population), ‘PPI’ (concept) and ‘stroke research’ (context). Closely examining these concepts and using thesaurus terms where appropriate generates a comprehensive list of search terms including stroke survivor, stroke carer, patient and public involvement, patient participation, patient engagement, consumer involvement, stakeholder participation and stroke research.

A sample of an indicative search strategy for the PubMed database is provided below ( Table 2).

**Table 2.** Sample search strategy.

**Population** – individuals with experience of stroke	
#1	(patient OR survivor OR adult OR family OR carer OR parent)
#2	(stroke OR poststroke OR post-stroke OR cerebrovascular disease OR cerebrovascular disorders OR CVD OR CVA OR brain infarction OR intracranial arterial diseases)
#3	#1 AND #2
**Concept** – PPI (patient and public involvement)	
#4	(“patient and public involvement” OR “patient involvement” OR “patient partnership” OR “patient collaboration” OR “patient engagement” OR “patient advocacy” OR “patient participation” OR “consumer participation” OR “consumer involvement” OR “consumer engagement” OR “stakeholder participation” OR “stakeholder engagement” OR “patient driven” OR “survivor participation” OR patient participation [MeSH] OR patient advocacy [MeSH] OR stakeholder participation” [MeSH] OR survivor participation)
**Context** – stroke research	
#5	(stroke OR poststroke OR post-stroke OR cerebrovascular disease OR cerebrovascular disorders OR CVD OR CVA OR brain infarction OR intracranial arterial diseases)
#6	(research OR review OR investigat* OR study OR project OR evaluation)
#7	#5 AND #6
#8	#3 AND #4 AND #7

### Stage 3: Study selection

Studies retrieved by the targeted search strategy will be collated and uploaded to Covidence for screening and final selection. Duplicates will be removed. Two reviewers will independently screen each record by title and abstract using the specified inclusion/exclusion criteria. Included studies following review of titles and abstract, will be retrieved in full text and again will be assessed independently by two reviewers. Disagreements will be resolved by discussion and where consensus is not achieved a third researcher will be consulted before final inclusion/exclusion.

### Stage 4: Charting the data

A data charting form will be devised to determine the relevant information to extract from the included sources using Microsoft EXCEL spreadsheet software. This will be developed in accordance with the JBI guidelines ^20^ for charting and extracting data and in conjunction with the purpose specific research cycle framework developed for this review. It is anticipated that this form will require review and modification as the process advances and familiarity with selected studies dictates a need to capture further information.

- Author(s)
- Year of publication
- Title
- Origin/country of origin
- Study aims/purpose
- Study type
- PPI concept description, approach, stages of inclusion
- PPI representation: population, profile, and underrepresentation of groups, where present
- PPI research activities, contributions
- Facilitators and barriers identified in incorporating PPI
- PPI in research evaluation, benefits, impact

### Stage 5: Collating, summarising and reporting the results

Data extraction will be completed independently by at least 2 reviewers and any discrepancies will be resolved by discussion to reach consensus. In accordance with scoping review guidelines, the quality of any study included in this review will not be assessed for risk of bias.

The review findings will be reported using the PRISMA-ScR guidelines ^21^. A PRISMA flow diagram will be produced to present an overview of the identification screening, eligibility and inclusion phases, with the reasons for exclusion of studies in the screening process provided.

The extracted data will be presented in tabular format, providing a brief summary of each individual study included in the review and documenting barriers/facilitators and impact of participation, where reported in individual studies. Data from the studies included in the review will be collated and mapped to the stage/s of the research cycle. Summary tables will be utilised to present the current volume, publication year, origin, study characteristics, and methodological design. The benefit of PPI in stroke research will be recorded where the study details changes (added value) in the research methods/activities; interpretation of results, dissemination activities or in policy/practice that was attributed to experience-based involvement. Impact of PPI in stroke research will be recorded where the individual study details changes in the research activity; interpretation of results, dissemination activities or in policy/practice that was attributed to PPI in stroke research. Evaluation of impact will be guided by appraisal criteria ^25^ which considers whether the paper discusses the difference PPI has made to the research activities and the impact of user involvement on the research project (e.g. length of study, financial implications of involvement activities, cost-benefit analyses), and whether benefits claimed are supported by examples from the research project.

PPI participants in current stroke research will be profiled and examined as representative of the stroke population and family/carer network. The diversity of the experience-based stroke research partners will be examined for sex, ethnicity, stroke type, disability level and will consider issues that may marginalise specific stroke groups from PPI activities including notable mobility and/or communication issues.

A compendium of approaches taken to PPI in stroke research will be developed including the involvement as described e.g. consultation, collaboration, and user-control across the different stages of the research cycle. A narrative synthesis will be conducted for findings that focus on the contribution made by PPI in stroke research and the methods used to report this in the scientific literature. Consistent with scoping review guidance, no appraisal of the quality of the studies will be conducted.

### Stage 6: Stakeholder consultation

Strong PPI engagement will be embedded in this scoping review methodology. A stroke research advisory panel including multiple stakeholders (people affected by stroke – survivors/carers/advocates, clinicians, research team) has been developed to guide both the design and the conduct of this review and further stroke research activities. A co-author on this study protocol has personal experience of stroke (JH)and acts as our stroke champion on all PPI issues, contributing to the research development. As an integral member of the review team our champion will collaborate in constructing the questions to drive data extraction, and / or reporting and disseminating the findings.

### Study status

Electronic database searches are in progress and will be completed by 1 ^st^ December 2021. No study selection process, formal screening or data extraction has commenced at time of submission.

## Discussion/conclusion

There is a growing consensus on the importance of PPI in health and social care research, although current practices in stroke research remain uncharted. A scoping review is indicated where the objective of the review is to generate a clear picture of a concept and its gaps in existing research ^26^. Gaps in current stroke research practices may be identified thereby providing some key opportunities and recommendations for future research. These broad objectives dictated our approach, as presented, to identifying and mapping the existing scientific literature that reports PPI in current stroke research.

While a scoping review can take a broader approach and search for opinion pieces, editorials and guideline documents, we opted in this review to keep a narrower focus to only stroke research with empirical outputs to capture current practices, as opposed to including and summarising best practice recommendations. Where present, PPI activity in stroke research may not always be explicitly reported in scientific papers and appropriately indexed and we acknowledge this potential barrier in searching the stroke research literature. We further acknowledge that while we chose to focus solely on research with empirical output/s, the quality of individual research studies included in the review and primary results unrelated to PPI are not commented on directly.

The findings from this scoping review will help identify what is currently reported in terms of the profile of PPI participants in stroke research, the strategies employed for collaboration and the PPI contributions across the phases of the research cycle. Knowledge gaps will be identified and future priorities for PPI in stroke research identified as a result of this scoping review. Dissemination of the findings will contribute to enhanced awareness and understanding of the need for PPI in stroke research as well as highlighting the current impact of PPI in stroke research, where reported.

## Data availability

### Underlying data

No data are associated with this article.

### Extended data

Open Science Framework: Patient and public involvement in stroke research: a scoping review protocol. https://doi.org/10.17605/OSF.IO/FP65E ^23^

This project contains the following extended data:

- PubMedSearchStrategy.docx
- PRISMA-ScR-Fillable-Checklist_11Sept2019.pdf

Data are available under the terms of the Creative Commons Zero "No rights reserved" data waiver (CC0 1.0 Public domain dedication).
