# Supplementary material for: Patient and public involvement in stroke research: a scoping review protocol
Source: HRB Open Res. 2022 Jul 28;4:118. Originally published 2021 Nov 8. [Version 2] doi: 10.12688/hrbopenres.13449.2 (PMC9345265; doi:10.12688/hrbopenres.13449.2)
Supplement: Peer review history

# Peer review: Reviewer response for version 2

**DOI:** 10.21956/hrbopenres.14854.r32584

**Published:** 2022-08-01

**Authors:** Elizabeth A Lynch

**Affiliations:** 1 Caring Futures Institute, College of Nursing and Health Sciences, Flinders University, Adelaide, SA, Australia

Great work - authors have responded and addressed all queries in full

Is the study design appropriate for the research question?

Yes

Is the rationale for, and objectives of, the study clearly described?

Yes

Are sufficient details of the methods provided to allow replication by others?

Partly

Are the datasets clearly presented in a useable and accessible format?

Not applicable

Reviewer Expertise:

Stroke, rehabilitation

I confirm that I have read this submission and believe that I have an appropriate level of expertise to confirm that it is of an acceptable scientific standard.

# Peer review: Reviewer response for version 1

**DOI:** 10.21956/hrbopenres.14657.r32067

**Published:** 2022-05-30

**Authors:** Julie Broderick

**Affiliations:** 1 Discipline of Physiotherapy, School of Medicine, Trinity College Dublin, University of Dublin, Dublin, Ireland

The authors need to be commended for a protocol in this valuable topic area of PPI applied to stroke. This emerging area very much lends itself to the scoping review methodology.  I particularly liked how PPI was the topic of this protocol and how it was integrated into Stage 6 of the Arksey and O Malley framework. I have some relatively minor points which are mainly around providing more detail around the scoping review protocol methodology. 

 Some points for your consideration: 

1. I would suggest that the aim is reframed as a single overarching protocol aim, rather than how it is numbered currently
2. This is more a formatting issue - but could the objectives be numbered
3. It is useful to refer to seminar papers in the area of scoping review methodology - Arksey and O' Malley and Levac which are included but I would also suggest referring to Chapter 11 of the JBI Manual for Evidence Synthesis as a methodological framework for this scoping review. I see the JBI data extraction tool was listed but this Chapter is worth reading in detail, also using it as a framework and referring to it in the context of scoping review methodology.
4. In stage 2: 'Leanus' (grey literature) is mentioned in the abstract and methodology - I think this should be spelt 'Lenus', Will reference list of included studies/sources of evidence be checked for possible inclusions. An indicative search strategy is included but could a full search strategy for one of the electronic databases be included as extended data. Could more detail of the grey literature search be included  - eg Cadth grey matters tool/others. 
5. in Stage 3: Could it be stated that reasons for exclusions will be recorded and charted in the PRISMA flow diagram
6. In Stage 4: Clarify if data will be extracted by one author and verified by another. Following the JBI convention, risk of bias assessment is not a necessity. Clarify whether a risk of bias assessment will take place or not. 
7. In Stage 5: Specify how impact will be evaluated/charted and how representativeness of participants will be assessed from an EDI perspective. 
8. Discussion: It is mentioned that a scoping review objective is to generate gaps in existing research - but gaps will not be explored in this review so this point may need revision in the context of this protocol.
9. Final point - Could PRISMA-P checklist with location of reporting elements be uploaded as extended data. 

Is the study design appropriate for the research question?

Yes

Is the rationale for, and objectives of, the study clearly described?

Yes

Are sufficient details of the methods provided to allow replication by others?

Yes

Are the datasets clearly presented in a useable and accessible format?

Partly

Reviewer Expertise:

Research in physical functioning across chronic diseases with a focus on socially excluded populations. Evidence synthesis.

I confirm that I have read this submission and believe that I have an appropriate level of expertise to confirm that it is of an acceptable scientific standard.

# Response

**Published:** 2022-07-18

**Authors:** Patricia Hall

**Affiliations:** UCD , Ireland

Thank you for your positive comments and detailed points for consideration.

 Please see below detailed responses:

**Point 1:** I would suggest that the aim is reframed as a single overarching protocol aim, rather than how it is numbered currently

**In response to Point 1** – Thank you, numbering has been removed as suggested.

 This now reads as below:

*The overarching aim of this review is to identify and describe current practices of PPI in stroke research. Specifically, this review will focus on the nature, design and type/s of studies that involve patients and/or members of the public in the planning, conduct and/or dissemination of stroke research, and explore how PPI has been conceptualised in stroke research.*

**Point 2**: This is more a formatting issue - but could the objectives be numbered

**In response to Point 2** – I have reformatted the objectives in a numbered sequence.

 This now reads as:

*Objectives:  *

1. *map the volume, type and range of PPI approaches in stroke research*
2. *profile PPI participants and examine representativeness of participants from an equality, diversity and inclusion (EDI) perspective *
3. *map PPI research activities against the stroke research cycle *
4. *collate reported enablers and barriers to PPI application in stroke research*
5. explore *the impact of PPI on stroke research, clinical practice and health policy*

*​​​​​​​* **Point 3 :** It is useful to refer to seminar papers in the area of scoping review methodology - Arksey and O' Malley and Levac which are included but I would also suggest referring to Chapter 11 of the JBI Manual for Evidence Synthesis as a methodological framework for this scoping review. I see the JBI data extraction tool was listed but this Chapter is worth reading in detail, also using it as a framework and and referring to it in the context of scoping review methodology.

**In response to Point 3** – thank you I have read this chapter in the JBI manual in detail and with great interest.

 This now reads in the manuscript:

*The review will conform to the 5 stages of the Arksey & O'Malley framework for scoping studies ^18^ refined by Levac ^19^ and the methodological framework described by* *Peters in the JBI Manual for Evidence Synthesis (Chapter 11). ^20^*

**Point 4:** In stage 2: 'Leanus' (grey literature) is mentioned in the abstract and methodology - I think this should be spelt 'Lenus', Will reference list of included studies/sources of evidence be checked for possible inclusions. An indicative search strategy is included but could a full search strategy for one of the electronic databases be included as extended data. Could more detail of the grey literature search be included  - eg Cadth grey matters tool/others.

**In response to Point 4 –** (i) apologies for the spelling error; this has now been corrected in the manuscript. (ii) Reference lists will be checked and sample PubMed search strategy is included as supplementary information. (iii) Thank you I was not familiar with Cadth but will now explore this grey literature checklist tool when searching the grey literature.

 The manuscript now reads:

*The search will involve the electronic databases PubMed, CINAHL, EMBASE, PsychINFO and the Cochrane Database of Systematic Reviews (Supplementary file). These* *libraries were chosen as they will yield a broad, cross disciplinary range of studies. In addition, reference lists of included studies will be checked. To target relevant grey literature, a systematic search will be conducted in the key databases, e.g. OpenGrey and Lenus. A grey literature checklist e.g. Cadth (Canada’s drug and health technology agency), will help ensure a comprehensive search is completed.*

**Point 5:** in Stage 3: Could it be stated that reasons for exclusions will be recorded and charted in the PRISMA flow diagram

**In response to Point 5 - **Thank you **, **I have included this detail in the manuscript which now reads:

*A PRISMA flow diagram will be produced to present an overview of the identification, screening, eligibility and inclusion phases, with  the reasons for exclusion of studies in  the screening process provided.*

**Point 6:** In Stage 4: Clarify if data will be extracted by one author and verified by another. Following the JBI convention, risk of bias assessment is not a necessity. Clarify whether a risk of bias assessment will take place or not.

**In response to Point 6 –** Thank you, I have clarified the extraction process and the manuscript now includes:

*Data extraction will be completed independently by at least 2 reviewers and any discrepancies will be resolved by discussion to reach consensus. In accordance with scoping review guidelines, the quality of any study included in this review will not be assessed for risk of bias.*

**Point 7:** In Stage 5: Specify how impact will be evaluated/charted and how representativeness of participants will be assessed from and EDI perspective.

**In response to Point 7 - **it is expected the process of summarising and reporting results will be an iterative process. Where individual studies provide detail, the impact will be recorded and the diversity of PPI partners will be examined. The manuscript now reads:

*The extracted data will be presented in tabular format, providing a brief summary of each individual study included in the review and documenting barriers/facilitators and impact of participation, where reported in individual studies. The benefit of PPI in stroke research will be recorded where the study details changes (added value) in research methods/activities; interpretation of results, dissemination activities or in policy/practice that was attributed to experience-based involvement. Evaluation of impact will be guided by appraisal criteria ^25 ^which considers whether the paper discusses the difference PPI has made to the research activities and the impact of user involvement on the research project (e.g. length of study, financial implications of involvement activities, cost-benefit analyses), and whether benefits claimed are supported by examples from the research project.*

*The diversity of the experience-based stroke research partners  will be examined for sex, ethnicity, stroke type, disability level and will consider issues that may marginalise specific stroke groups from PPI activities including  notable mobility and/or communication issues.*

**Point 8:** Discussion: It is mentioned that a scoping review objective is to generate gaps in existing research - but gaps will not be explored in this review so this point may need revision in the context of this protocol.

**In response to Point 8 –** apologies it was not the intention to suggest gaps will be explored but rather by comprehensively mapping current stroke research practice, gaps may be identified thereby providing some key opportunities for future research. 

 This is now included in the manuscript as:

*Gaps in current stroke research practices may be identified thereby providing some key opportunities and recommendations for future research.*

**Point 9:** Final point - Could PRISMA-P checklist with location of reporting elements be uploaded as extended data.

**In response to Point 9 -** A checklist is now included in a supplemental information file

# Peer review: Reviewer response for version 1

**DOI:** 10.21956/hrbopenres.14657.r30802

**Published:** 2021-11-22

**Authors:** Elizabeth A Lynch

**Affiliations:** 1 Caring Futures Institute, College of Nursing and Health Sciences, Flinders University, Adelaide, SA, Australia

This scoping review will present the state of play regarding PPI in stroke research, which is an exciting concept.

- In introduction, I would be tempted to provide more hard data/numbers in statements in the first paragraph  - e.g. "many survivors" (give a number or proportion), "significant increase" (from what level) etc.
- With regard to the inclusion criteria, I think there needs more detail re "Studies with a clear statement in relation to PPI activities/ initiatives which fit the principles of PPI in stroke research, irrespective of terminology" - does this need to be presented in the title? Or abstract? Or paper? Do the authors envisage reading every abstract from the initial search (which is likely to be huge) to look for PPI? Or look for this in titles which might be more feasible but more likely to miss relevant publications.
- "Strong PPI engagement will be embedded in this scoping review methodology... A PPI Champion / stroke survivor contributed to the research objectives and the refining of the search strategy described in this protocol" - can you be explicit whether there is someone with lived experience of stroke in the authorship team? I think there is someone but I'm not quite sure (and if there isn't, should there be, to ensure/demonstrate strong PPI engagement?)

Is the study design appropriate for the research question?

Yes

Is the rationale for, and objectives of, the study clearly described?

Yes

Are sufficient details of the methods provided to allow replication by others?

Partly

Are the datasets clearly presented in a useable and accessible format?

Not applicable

Reviewer Expertise:

Stroke, rehabilitation,

I confirm that I have read this submission and believe that I have an appropriate level of expertise to confirm that it is of an acceptable scientific standard, however I have significant reservations, as outlined above.

# Response

**Published:** 2022-07-18

**Authors:** Patricia Hall

**Affiliations:** UCD , Ireland

Thank you for your positive comments and detailed feedback.

 Please see detailed responses below:

**Point 1:** In introduction, I would be tempted to provide more hard data/numbers in statements in the first paragraph  - e.g. "many survivors" (give a number or proportion), "significant increase" (from what level) etc.

**In response to Point 1** – in the introduction I have provided  further  data on specific numbers relating to Ireland and Europe.

 This now reads:

*Stroke is a major cause of death and disability worldwide* *^1^* *. Globally stroke is the second leading cause of death and third leading cause of disease burden accounting for almost 5% of disability-adjusted life  years* *According to the latest National Audit of Stroke in Ireland (NOCA), almost 6,000 people were admitted to hospital in 2019 and 71% of those with ischaemic stroke had disabilities on discharge.*

*Despite advances in prevention, early recognition/diagnosis and treatment, projections indicate a significant increase in stroke events worldwide in the coming years. ^5^ An ageing population contributes strongly to this increase with  European predictions estimating a 34% rise in stroke cases by 2035.*

**Point 2.** With regard to the inclusion criteria, I think there needs more detail re "Studies with a clear statement in relation to PPI activities/ initiatives which fit the principles of PPI in stroke research, irrespective of terminology" - does this need to be presented in the title? Or abstract? Or paper? Do the authors envisage reading every abstract from the initial search (which is likely to be huge) to look for PPI? Or look for this in titles which might be more feasible but more likely to miss relevant publications.

**In response to Point 2** – I have clarified the inclusion criteria to make the anticipated screening process more explicit. We do not think it will be possible to identify PPI activity in stroke research only in the title and abstract. We now detail that an inclusive approach will be taken whereby if not identified by title and abstract, full manuscript review will be undertaken with a primary focus on the methodology and acknowledgement sections. This will indeed be potentially onerous but in order to be as comprehensive as possible, since PPI is often not called out in the title/abstract, we feel it is a necessary undertaking to fully address the question. However as this approach will likely generate a high volume of manuscripts to review, a publication date cut-off of 2014 will now be applied to include only studies published after the first handbook of PPI was developed for health researchers.

 The inclusion criteria now states: 

*Studies with a clear statement in relation to PPI activities/initiatives which fit the principles of PPI in stroke research, irrespective of terminology. An inclusive approach to screening will be taken whereby if PPI is not mentioned in the title or abstract, the full text manuscript will be reviewed with particular focus on the methods and acknowledgements sections. As PPI is a relatively new concept a publication date cut-off of 2014 will be applied to include only studies published after the first handbook was developed for health researchers *was published ^22^

# Response

**Published:** 2022-07-18

**Authors:** Patricia Hall

**Affiliations:** UCD , Ireland

**Point 3 : **"Strong PPI engagement will be embedded in this scoping review methodology... A PPI Champion / stroke survivor contributed to the research objectives and the refining of the search strategy described in this protocol" - can you be explicit whether there is someone with lived experience of stroke in the authorship team? I think there is someone but I'm not quite sure (and if there isn't, should there be, to ensure/demonstrate strong PPI engagement?)

**In response to Point 3** – apologies I did not make it clear that one of the authors has indeed the lived experience of stroke. I have corrected this omission as it is very important to us to demonstrate our strongly embedded PPI.

 This now reads in the manuscript:

*A co-author on this study protocol has personal experience of stroke (JH)and acts as our stroke champion on all PPI issues, contributing to the research development.*
